# Supplementary material for: New Preservative-Free Formulation for the Enhanced Ocular Bioavailability of Prostaglandin Analogues in Glaucoma
Source: Pharmaceutics. 2022 Feb 20;14(2):453. doi: 10.3390/pharmaceutics14020453 (PMC8877962; doi:10.3390/pharmaceutics14020453)
Supplement: Supplementary file 1 [file pharmaceutics-14-00453-s001.zip › pharmaceutics-1591017-supplementary.pdf]

Supplementary Material

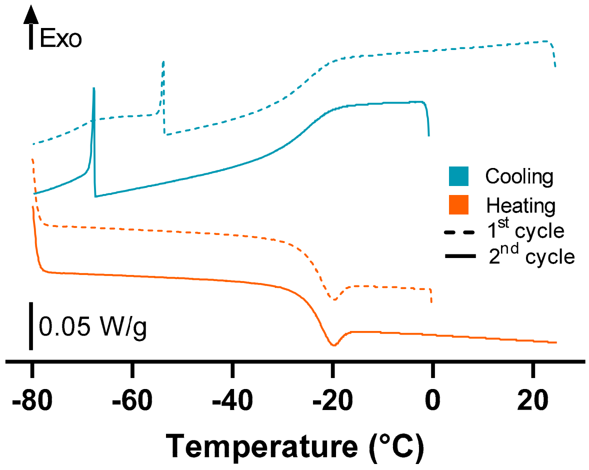

Figure S1. DSC thermograms of two cooling/heating cycles performed on the same travoprost sample, displaying thermal reversibility of the event of interest (Cooling/Heating rate of 5 °C/min, before heating the sample was annealed at −80 °C for 3 minutes). Displayed thermograms were shifted for clarity.

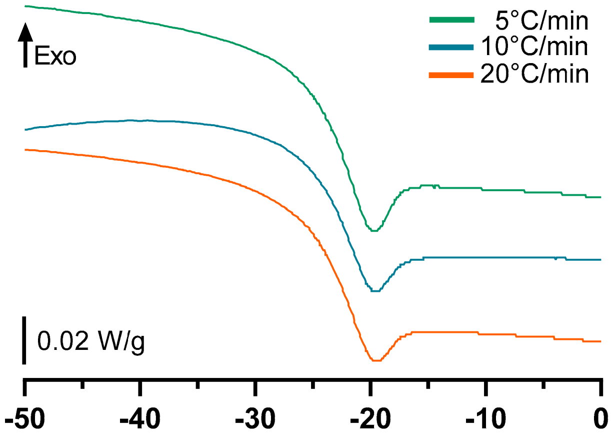

Figure S2. DSC Thermograms focusing on the glass transition with relaxation event displayed when heating travoprost at a 5 °C/min after a cooling at 5, 10 or 20 °C/min from ambient temperature to −80 °C followed by an annealing at −80°C for 3 minutes. Displayed thermograms were shifted for clarity.

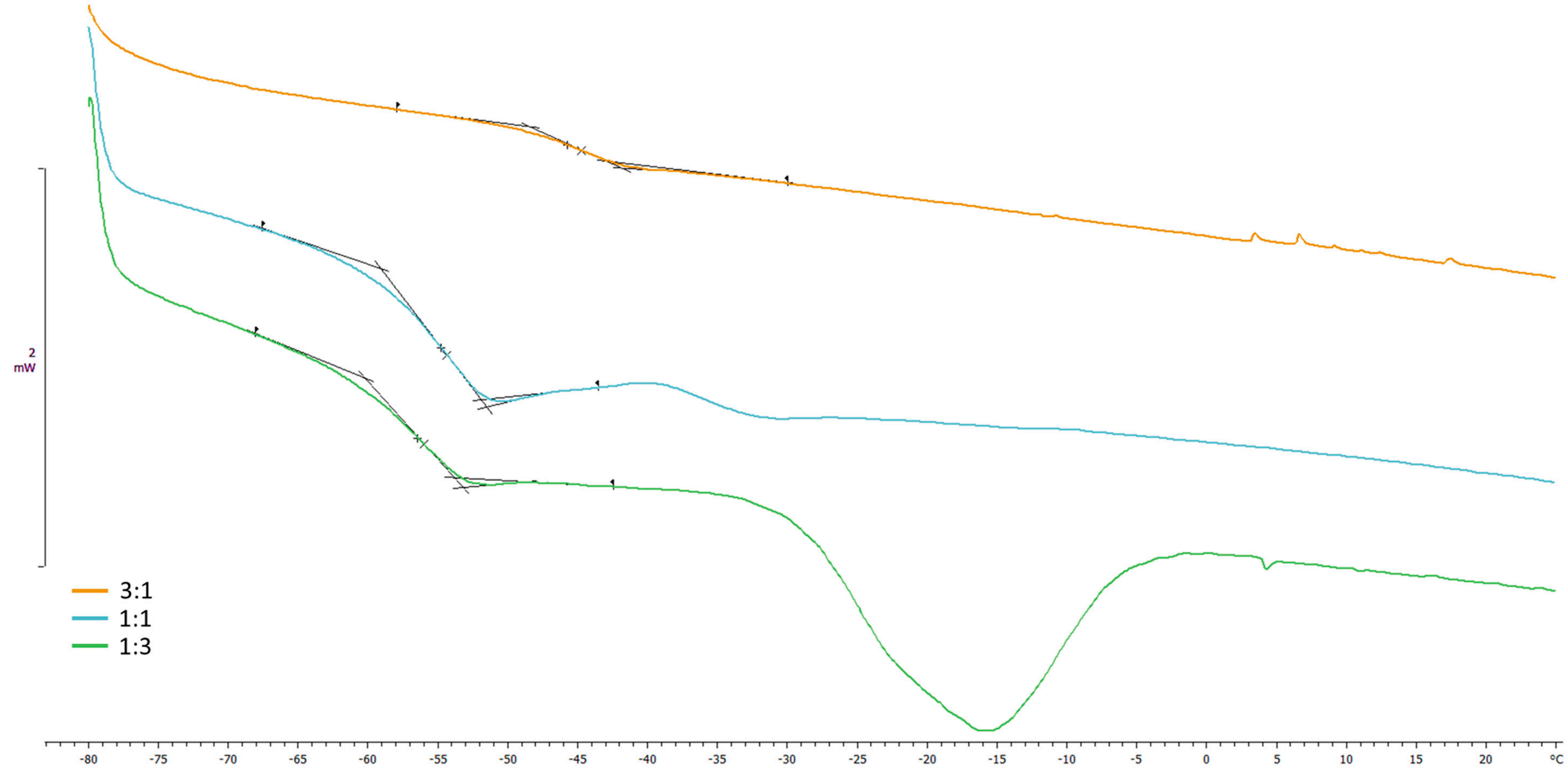

Figure S3 Illustration of thermal event integration applying the "parallel tangents method" to thermograms of different travoprost:PS80 weight ratios, namely 3:1 (top curve), 1:1 (middle curve) and 1:3 (bottom curve).
